# Effects of High-Pressure Homogenization on Pectin Structure and Cloud Stability of Not-From-Concentrate Orange Juice

**DOI:** 10.3389/fnut.2021.647748

**Published:** 2021-05-05

**Authors:** Wantong Yu, Jiefen Cui, Shaojie Zhao, Liping Feng, Yanqi Wang, Junmei Liu, Jinkai Zheng

**Affiliations:** ^1^College of Food Science and Engineering, Jilin Agricultural University, Changchun, China; ^2^Institute of Food Science and Technology, Chinese Academy of Agricultural Sciences, Beijing, China

**Keywords:** high-pressure homogenization, NFC orange juice, pectin, structure, stability

## Abstract

Not-from-concentrate (NFC) juice is popular with consumers due to its similarity to fresh fruit juice in taste, flavor, and beneficial nutrients. As a commonly used technology in fruit juice production, high-pressure homogenization (HPH) can enhance the commercial value of juice by improving the color, flavor, taste, and nutrient contents. In this study, the effects of HPH on the pectin structural properties and stability of NFC orange juice were investigated. The correlations between HPH-induced changes in the structure of pectin and the stability of orange juice were revealed. Compared with non-homogenized orange juice, HPH increased the galacturonic acid (GalA) content and the linearity of pectin, while decreasing the molecular weight (Mw), pectin branching, and rhamnogalacturonan (RG) contribution, and cracks and pores of different sizes formed on the surface of pectin, implying depolymerization. Meanwhile, with increasing pressure and number homogenization of passes, HPH effectively improved the stability of NFC orange juice. HPH can effectively prevent the stratification of orange juice, thereby promoting consumer acceptance and endowing a higher commercial value. The improvement of the stability of NFC orange juice by HPH was related to the structural properties of pectin. Turbidity was significantly (*P* < 0.01) positively correlated with GalA and pectin linearity, but was significantly (*P* < 0.01) negatively correlated with Mw, RG contribution, and pectin branching. Modification of pectin structure can improve the stability of NFC orange juice. In this work, the relationship between the pectin structure and stability of NFC orange juice is elucidated, providing a path toward improving consumer acceptance and enhancing the palatability and nutritional and functional qualities of orange juice. Manufacturers can use this relationship to modify pectin directionally and produce high-quality NFC orange juice beverages.

## Introduction

The orange is one of the most important agricultural products, with high annual yield worldwide (73.3 million tons in 2019), much of which is used to produce orange juice (1). In recent years, not-from-concentrate (NFC) orange juice has become increasingly popular among consumers because its flavor is closer to that of freshly squeezed orange juice (2, 3). More importantly, NFC orange juice is rich in various nutrients (pectin, vitamin C, carotenoids, and flavanones) and exhibits several biological activities (4), including antioxidant (5), anti-inflammatory (6), and anticarcinogenic effects (7). It is processed from fresh oranges by washing, squeezing, homogenizing, and sterilizing. It is not concentrated during its production process and no chemical preservatives are added (3). The endocarp cells are ruptured during the process of mechanical squeezing, and the membrane as well as many components (pectin, protein, cellulose, hemicelluloses, and hesperidin) are suspended in the orange juice (8, 9). These suspensions lead to the cloudy and turbid appearance of NFC orange juice and affect its cloud stability. Due to containing particles of different sizes and the existence of pectin and pectin methylesterase (PME), orange juice is easily delaminated (10), which destroys its stability and thus reduces its sensory and nutritional properties, ultimately affecting consumer acceptance and the market value of orange juice. Therefore, cloud stability is an important indicator of the food, nutritional, and functional qualities of orange juice.

Pectin is part of the cloud material and plays a vital role in cloud stability. Pectin in orange juice is transformed by PME from high-methoxyl pectin to low-methoxyl pectin and pectic acid, which forms insoluble complexes with calcium ions and leads to decreased cloud stability (11), which in turn affects the sensory and nutritional properties of orange juice. Pectin is a complex polysaccharide, and its structure is mainly composed of three domains: homogalacturonan (HG), rhamnogalacturonan-I (RG-I), and rhamnogalacturonan-II (RG-II) (12, 13). HG, also known as the “smooth zone,” is composed of 1,4-linked α-D-galacturonic acid (α-D-GalA) residues, which are considered the backbone of pectin, with some C-6 carboxyl groups being methylated. The functional properties of pectin in fruit-based products depend to a large extent on the properties of the HG region. RG-I comprises a backbone of alternating α-L-rhamnose and α-D-GalA residues. To most of rhamnose (Rha) residues side chains at C4 are attached that consist mainly of arabinose (Ara) and galactose (Gal) (4). RG-II has a short 1,4-linked α-D-GalA backbone, and its side chain is composed of 12 different types of sugars (12). Generally, the properties of pectin are influenced by galacturonic acid (GalA), molecular weight (Mw), and neutral sugars (14, 15). The proportion of esterified GalA residues and the distribution of esterified residues in the HG region strongly affect the solubility, thickening, gelling, and hydration properties of pectin (12). The Mw distribution of pectin is a major parameter that directly affects the apparent viscosity and other physicochemical properties (16). Notably, the structural characteristics of pectin are easily affected by the processing process, which further affects its physicochemical properties, functions, and applications (17).

High-pressure homogenization (HPH) is an important component of juice processing that can improve the stability and quality of the juice and release bioactive substances, thereby enhancing the nutritional and functional properties of the juice. HPH is realized by a piston forcing fluid through small holes. When the fluid passes through the valve, the rapid change of velocity and pressure produce turbulence and cavitation, fragmenting the polymer, particles, or cells in the system (11). HPH can enhance the turbidity and overall color of orange juice, reduce particle size, and increase the nutrient accessibility (total carotenoids, flavonoids, and vitamin C) and functional properties (antioxidant activity) in orange juice (18–20), which in turn increases consumer acceptance and improves the market value. For mixed juice (carrot, apple, and peach), HPH can increase the linearity of pectin and reduce the branches, Mw, and GalA content of pectin, indicating that pectin is depolymerised by HPH. Meanwhile, HPH can effectively improve the stability and increase nutrient contents in mixed juice (21, 22). Moreover, for carrot juice, HPH enhances the cloud stability, juice stability, and GalA content of pectin, and decreases the acetylation and Mw of pectin compared to non-homogenized (NH) juice. The effect of HPH on the structure of pectin further improves the bioavailability of carotenoids in carrot juice and the nutritional quality of the juice (13). HPH can also depolymerise commercial citrus pectin, as evidenced by a decrease in Mw (23). HPH can change the structure of pectin and improve the cloud stability of the juice, which in turn affects the sensory, nutritional, and functional properties of the juice and increases its commercial value.

However, the mechanism by which HPH modifies the pectin structure of NFC orange juice and the correlation between the stability of NFC juice and pectin structure remain unclear. Here, the effects of HPH treatments on the pectin structural properties and stability of NFC orange juice were investigated. Furthermore, the correlation between the modification of pectin structure and the stability of NFC orange juice was also elucidated. This work will provide theoretical guidance for the production of NFC orange juice of high nutritional and organoleptic quality, thereby improving the commercial value of orange juice.

## Materials and Methods

### Materials

Sunkist navel oranges (*Citrus sinensis* Osb. var. *brasliliensis* Tanaka) were obtained from the local market (Beijing, China), and were stored at 4°C until experiments. Sodium hydroxide, hydrochloric acid, 95% ethanol and trifluoroacetic acid (TFA) were bought from Shanghai Aladdin Biochemical Technology Co., Ltd. (Shanghai, China). Proteinase K was purchased from Beijing Solarbio Technology Co., Ltd. (Beijing, China). Monosaccharide standards and galacturonic acid (GalA) were purchased from Sigma–Aldrich (Shanghai, China). All the other chemicals and reagents were analytical grade.

### Preparation for NFC Orange Juice and High-Pressure Treatment

The oranges were washed, peeled and the pulp was squeezed into an electric juicer (MJ-WJS1222F, Midea, China) to crush the oranges. The pectin was extracted from the peel and added to the juice using the acid-extraction method to reduce the waste of resources. The crude juice was preliminarily refined by a high-speed homogenization (ULTRA-TURRAX T25 digital, IKA, Germany) and immediately filtered through a 100-mesh sieve to remove the impurities. Immediately after filtration, the orange juice was subjected to HPH.

HPH was carried out in a high-pressure homogenizer (APV-2000, SPX, Germany). Non-homogenized (NH) orange juice was used as the control group. The orange juice was divided into two equal parts. One part was subjected to HPH treatment at pressures of 30, 50, 100, and 150 MPa. The other part was subjected to 1, 2, and 3 homogenization passes at 100 MPa. The homogenizer was connected to the cooling circulation system. The temperature was kept constant at 25°C.

### Pectin Analysis

#### Pectin Extraction

To investigate the effects of HPH on the structural characteristics of pectin in NFC orange juice, pectin extracted from NH orange juice was used as the control group, and pectin extracted from orange juice treated with different HPH (homogenization pressure: 30 MPa, 50 MPa, 100 MPa and 150 MPa; homogenization passes: 1pass, 2 passes and 3 passes) was used as the experimental group. Pectin in NFC orange juice was extracted by slightly modified acid extraction and alcohol precipitation method (24). Briefly, NFC orange juice was adjusted to pH 2 with hydrochloric acid. The solution was heated at 90°C for 1 h with continuous stirring. The slurry was then centrifuged, and the obtained supernatant was precipitated with 95% ethanol (1:2, v/v). The precipitate was re-suspended in deionized water, enzymatically purified with proteinase K (Solarbio, Beijing, China), incubated, centrifuged, and dialyzed in the supernatant (MWCO: 6,000–8,000 Da). The dialysis process lasted for 24 h and the deionized water was replaced every 6 h. After dialysis, the supernatant was precipitated by the addition of three volumes of 95% ethanol, and the resulting precipitate was dried overnight at 40°C, and then ground to a powder.

#### Determination of Mw

High-performance size-exclusion chromatography coupled to multiangle laser light scattering (Dawn Heleos II; Wyatt Technology, Goleta, CA, USA), reflective index (RI) detector (Optilab rEX, WyAtt, USA) and ultraviolet (UV) detector (L-2400, Hitachi, Japan) were combined to determine the Mw of the extracted pectin. In detail, pectin (10 mg) was dissolved in eluent (0.1 M NaCl) overnight, then filtered through a 0.45-μm filter membrane before analysis. Samples (200 μL) were injected into a TSKgel column (TSKgel G4000PWxl; Tosoh Bioscience, King of Prussia, PA, USA). Elution was performed with 0.1 M NaCl solution at a flow rate of 0.5 mL/min for 30 min and the column was maintained at 35°C. The refractive index increment dn/dc was set to 0.135 mL/g.

#### Determination of Monosaccharide Composition

High-performance anion-exchange chromatography was achieved using an ICS-3000 Ion Chromatography System (Dionex, Sunnyvale, CA, USA) equipped with a CarboPac™ PA20 column and a pulsed amperometric detector. Pectin (10 mg) was hydrolyzed using 2 M trifluoroacetic acid for 1 h at 120°C. After cooling and nitrogen drying, samples were adjusted to 5 mL, diluted, and filtered through a 0.2-μm membrane. Mixtures of GalA, Gal, Ara, Rha, xylose, and glucose at different concentrations (0.01–5 mg/L) were used as standards. 10 μL samples were injected into ultra-pure water (A), 0.25 M NaOH (B) and 1 M NaAC (C) at a flow rate of 0.5 mL/min through a gradient elution procedure. The gradient elution procedure was as follows: 0–20 min: 94% A and 6% B; 20–20.1 min: 89% A, 6% B and 5% C; 20.1–35 min: 74% A, 6% B and 20% C; 35.1–45 min: 20% A and 80% B; 45.1–55 min: 94% A and 6% B.

#### Determination of the Degree of Methylation (DM)

The DM of pectin was determined by titration (25). Extracted pectin (0.2 g) was dissolved in 20 mL of distilled water and stirred at room temperature for 2 h. Three drops of phenolphthalein indicator were added, and the mixture was titrated with 0.1 M NaOH until the color of the indicator changed to pink, and then the volume (V_1_) was recorded. Next, 2 mL 0.25 mol/L NaOH was added to the solution, and 2 mL 0.25 mol/L hydrochloric acid solution was added after stirring at 25°C for 30 min to neutralize the excess NaOH. The solution was titrated with 0.1 M NaOH until the color of the indicator changed back to pink, then the volume (V_2_) was recorded. The DM of pectin was obtained according to Equation (1).

(1) $$\begin{array}{l} \text{DM }\left(\%\right)=\frac{{\text{V}}_{2}}{{\text{V}}_{1}+\;{\text{V}}_{2}}\;\times \;100 \end{array}$$

#### Scanning Electron Microscopy (SEM) Analysis

SEM (SU8010; Hitachi, Tokyo, Japan) was used to observe the surface morphology of pectin extracted from orange juice after different treatments (homogenization pressure: 30, 50, 100, and 150 MPa; homogenization passes: 1pass, 2 passes, and 3 passes). Pectin powder samples (5 mg) were fixed to the sample table with double-sided tape, and each sample was coated with gold powder under vacuum conditions (26). Images were taken at an acceleration voltage of 10 kV at 3000× magnification.

### Stability Analysis of NFC Orange Juice

#### Turbidity and Pulp Sedimentation

The turbidity of raw NFC orange juice was determined after centrifugation at 25°C and 3,000× g for 10 min.

NFC orange juice (10 mL) was stored at 4°C for 6 days for the determination of the pulp sedimentation (27). The sedimentation index (IS) of NFC orange juice was estimated using Equation (2). Where V_s_ was the sedimentation volume of juice (mL); V_t_ was the total volume of juice (mL).

(2) $$\begin{array}{l} \text{IS }\left(\%\right)=\left(\frac{{\text{V}}_{\text{s}}}{{\text{V}}_{\text{t}}}\right)\;\times 100 \end{array}$$

#### Particle Size Determination

The particle size parameters were determined using a Mastersizer 2000 (Malvern Instruments, Malvern, UK). Laser diffraction was used to measure particles from 0.02 μm to 2,000 μm. The median particle diameter (D_50_, μm), volume-based diameter (D[4,3], μm), and area-based diameter (D[3,2], μm) were calculated using the software (Microtrac-Bluewave, Montgomeryville, PA, USA) provided with the equipment.

#### Viscosity Curves

Viscosity measurements of NFC orange juice samples were carried out using a rheometer (Physica MCR 301; Anton Paar, Graz, Austria) equipped with parallel plates (50-mm diameter) with a gap size of 1 mm. The temperature was maintained constant at 25°C using a Peltier system. In a steady-state shear mode, NFC orange juice samples (2.3 mL) were placed between the two plates and the shear rate was increased from 0.01 to 300 s^−1^ (28).

#### PME Activity

NFC orange juice (20 mL) was added to 40 mL of 2 M NaCl solution containing 1% pectin. The pH of the pectin–juice mixture was adjusted to 7 using 1 M NaOH. After pH stabilization, 1 mL of 0.05 M NaOH was added, and the time required to restore the pH to 7 was measured. Enzyme activity was calculated according to equation 3. Values were converted to percent residual activity relative to the untreated control sample. The NH orange juice represented 100% PME activity.

(3) $$\begin{array}{l} \text{PME activity }\left(\text{unit}/\text{mL}\right)\\ \;=\frac{\left(\text{mL NaOH}\right)\;\times \;\left(\text{NaOH normality}\right)\;\times \;10^{3}}{\left(\text{minutes}\right)\;\times \;\left(\text{mL NFC orange juice}\right)} \end{array}$$

### Simulation Verification Experiment

Oranges were washed, peeled, juiced, and immediately filtered through a 100-mesh sieve to remove pulp using an electric juicer. The filtered orange juice was not treated with HPH. Instead, pectin solution was treated with 100 MPa of HPH, then the homogenized pectin was added to the NFC orange juice at different concentrations (0, 0.015, 0.075, 0.375, and 1.875%). In addition, the Mw and monosaccharide composition of the pectin solution (0.075%) after homogenization were determined according to the methods in sections Determination of Mw and Determination of Monosaccharide Composition, respectively.

### Statistical Analysis

Data were analyzed using a one-way analysis of variance (ANOVA) following by LSD multiple comparisons or correlation analysis (SPSS 19.0 software) at the significance level of *P* < 0.05. The results were expressed as the average ± standard deviation. All assays were performed in triplicate. The figures were plotted using Origin 8.0 software.

## Results and Discussion

### Effect of HPH on the Pectin Structure

#### Mw and DM

To determine the mechanisms by which HPH improves the stability of NFC orange juice, pectin was extracted from homogenized orange juice, and the structural properties were determined. The Mw and DM results for pectin were shown in Table 1. The Mw of homogenized pectin (185–593 kDa) was considerably less than that of NH pectin (931 kDa). The Mw decreased from 593 to 250 kDa with increasing homogenization pressure. By increasing the number of homogenization passes, the Mw decreased from 262 to 185 kDa. Depolymerisation is the main change in polysaccharides during the HPH process (16). Compared with the homogenization pressure treatment, the number of homogenization passes had a more obvious effect on the depolymerization of pectin. Citrus pectin is poor in neutral sugar side chains. High pressure disrupts neutral sugar side chains of pectin, leading to a decrease in Mw (23, 29). The results were consistent with those of water-soluble pectin extracted from carrot juice as well as those of pectin extracted from potato, both of which showed a lower Mw of HPH-treated pectin compared to untreated pectin (13, 30). The relatively low Mw may improve water solubility, which in turn may promote biological activity and thus increase the nutritional quality of orange juice (4). It has been shown that high-pressure treatment leads to a decrease in pectin Mw and viscosity, thus indicating that the decrease in viscosity of NFC orange juice is associated with a decrease in pectin Mw (31). Compared to NH pectin, the DM gradually decreased with increasing homogenization pressure and the number of passes. Relatively low-DM pectin can improve the accessibility of carotenoids in juices (13). The DM ranged from 75.8 to 78.2%, with little change under different homogenization pressures or numbers of passes. This result shows that the DM of pectin in NFC orange juice is not affected by HPH. The DM values of HPH-treated citrus pectin and apple pectin were similarly unaffected (23). Thus, the changes in Mw after homogenization were not caused by the net change in electrostatic repulsion between the pectin molecules, which may improve total carotenoid bioaccessibility in NFC orange juice.

**Table 1:** Characteristic indicators of pectin in NFC orange juice.

	**Homogenization press (1 pass)**					**Homogenization pass (100 MPa)**			
	**NH**	**30 Mpa**	**50 Mpa**	**100 Mpa**	**150 Mpa**	**NH**	**1**	**2**	**3**
GalA (mol%)	62.97 ± 0.48^e^	66.25 ± 0.61^d^	67.71 ± 0.87^d^	72.86 ± 0.41^c^	75.07 ± 1.01^ab^	62.97 ± 0.48^e^	72.86 ± 0.41^c^	74.45 ± 0.01^b^	76.23 ± 0.74^a^
DM (%)	86.67 ± 0.71^a^	78.24 ± 1.10^b^	76.33 ± 0.17^b^	76.24 ± 0.13^b^	75.67 ± 0.04^b^	86.67 ± 0.71^a^	76.24 ± 0.13^b^	75.82 ± 0.44^b^	76.99 ± 0.72^b^
Fuc (mol%)	0.20 ± 0.03^dc^	0.41 ± 0.06^a^	0.40 ± 0.07^a^	0.24 ± 0.04^b^	0.12 ± 0.03^c^	0.20 ± 0.03^dc^	0.24 ± 0.04^b^	0.20 ± 0.01^bc^	0.35 ± 0.02^ab^
Rha (mol%)	2.12 ± 0.03^b^	2.15 ± 0.05^b^	2.16 ± 0.04^b^	2.14 ± 0.06^b^	2.08 ± 0.08^b^	2.12 ± 0.03^b^	2.14 ± 0.06^b^	2.07 ± 0.03^b^	2.38 ± 0.01^a^
Ara (mol%)	10.18 ± 0.18^a^	9.80 ± 0.22^ab^	9.49 ± 0.07^b^	8.26 ± 0.14^c^	7.55 ± 0.07^d^	10.18 ± 0.18^a^	8.26 ± 0.14^c^	8.58 ± 0.25^c^	9.59 ± 0.21^b^
Gal (mol%)	21.16 ± 0.43^a^	18.81 ± 0.31^b^	16.69 ± 0.27^c^	13.83 ± 0.31^d^	13.17 ± 0.29^e^	21.16 ± 0.43^a^	13.83 ± 0.31^c^	12.59 ± 0.47^e^	9.72 ± 0.53^f^
Glc (mol%)	2.95 ± 0.12^a^	2.14 ± 0.07^b^	3.05 ± 0.06^a^	2.27 ± 0.04^b^	1.44 ± 0.03^d^	2.95 ± 0.12^a^	2.27 ± 0.04^b^	1.65 ± 0.01^c^	1.11 ± 0.08^e^
Xyl (mol%)	0.42 ± 0.01^c^	0.44 ± 0.06^bc^	0.50 ± 0.07^b^	0.42 ± 0.01^c^	0.47 ± 0.02^bc^	0.42 ± 0.01^c^	0.42 ± 0.01^c^	0.46 ± 0.02^bc^	0.6 ± 0.01^a^
GalA/(Gal+Ara+Rha)	1.88 ± 0.03^f^	2.15 ± 0.09^e^	2.39 ± 0.01^d^	3.01 ± 0.05^c^	3.29 ± 0.10^b^	1.88 ± 0.03^f^	3.01 ± 0.05^c^	3.20 ± 0.06^b^	3.51 ± 0.07^a^
Rha/GalA	0.0337 ± 0.0001^a^	0.0325 ± 0.0002^b^	0.0312 ± 0.0007^c^	0.0293 ± 0.0006^d^	0.0277 ± 0.0010^e^	0.0337 ± 0.0001^a^	0.0293 ± 0.0006^d^	0.0278 ± 0.0001^e^	0.0264 ± 0.0003^f^
(Gal+Ara)/Rha	14.75 ± 0.04^a^	13.29 ± 0.01^b^	12.13 ± 0.03^c^	10.34 ± 0.02^d^	9.97 ± 0.07^e^	14.75 ± 0.04^a^	10.34 ± 0.09^d^	10.24 ± 0.01^d^	8.11 ± 0.01^f^
Mw ( ×10^5^ Da)	9.31 ± 0.01^a^	5.93 ± 0.07^b^	3.95 ± 0.02^c^	2.62 ± 0.04^d^	2.50 ± 0.02^e^	9.31 ± 0.01^a^	2.62 ± 0.04^d^	2.25 ± 0.09^f^	1.85 ± 0.03^g^

*Different superscript letters indicate significant differences among groups (P < 0.05)*.

#### Monosaccharide Composition

The monosaccharide compositions of pectin after different HPH treatments are shown in Table 1. The monosaccharide composition of pectin in NFC orange juice mainly consisted of GalA (63.0–76.2%), followed by Gal (9.7–21.2%), Ara (8.3–10.2%), and Rha (2.1–2.4%). Among them, GalA was derived from the HG and RG-I main chains. Rha was derived from the RG-I main chain, while Gal and Ara were derived from the RG-I side chain (32). The molecular structure of pectin in NFC orange juice is further characterized by the ratio of neutral sugars. GalA/(Gal+Ara+Rha) represents the linearity of the pectin. Rha/GalA represents the contribution of the RG region to the overall pectin. (Ara+Gal)/Rha is a measure of the degree of RG-I branching by comparing the amounts of RG-I side chain sugars (Ara and Gal) and Rha (33). The GalA content of homogenized pectin was higher than that of NH pectin and increased with pressure. Increasing the homogenizing pressure and the number of passes increased GalA/(Gal+Ara+Rha) and decreased Rha/GalA and (Gal+Ara)/Rha contents of the pectin. Compared with the homogenization pressure treatment, the number of homogenization passes resulted in more depolymerization of pectin side chains in NFC orange juice. The results show that HPH treatment enhanced the linearity of the pectin in NFC orange juice, reduced the contribution of RG to the overall pectin, and depolymerized the RG-I side chains. Therefore, it can be further clarified that the decrease in pectin Mw caused by HPH treatment is attributable to glycosidic bond cleavage. Higher linearity and lower branching of the pectin facilitate non-covalent hydrophobic interactions of the serum phase, thus improving the stability of orange juice (22).

#### SEM Analysis

**Figure 1:**
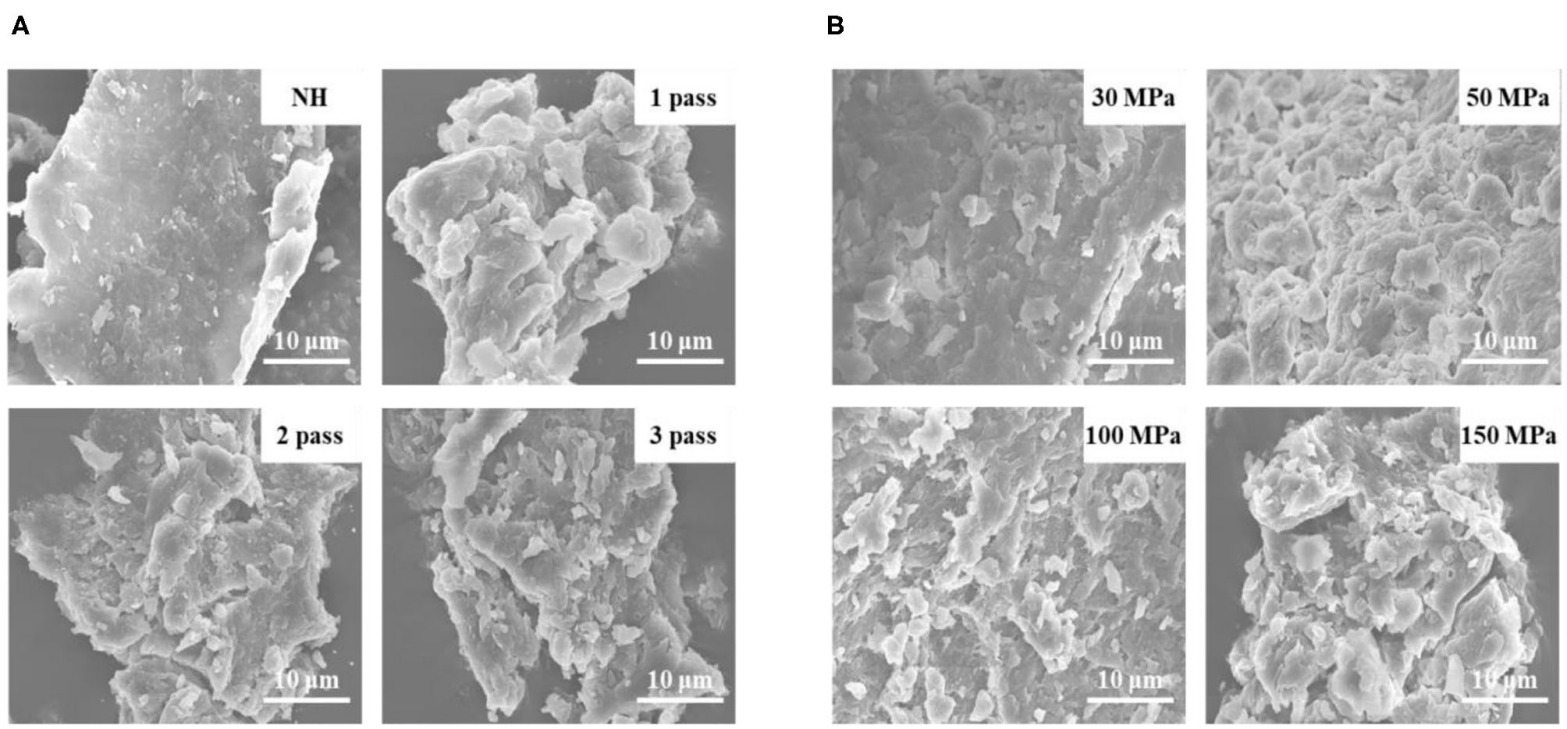
SEM images of NFC orange juice pectin after different HPH treatments (3000× magnification). **(A)** Homogenization pass; **(B)** Homogenization pressure.

The surface structure of pectin was examined by SEM. SEM micrographs of pectin treated with different HPH parameters are shown in Figure 1. The NH pectin was relatively tidy, with a lamella structure, and the surface was relatively smooth and compact. By increasing the number of homogenization passes from 1 to 3, the degree of surface cracking and pore size of pectin gradually increased (Figure 1A). After HPH under different pressures, the surface of pectin began to gradually become rough, and became extremely uneven at 50 MPa. When the pressure was further increased (100–150 MPa), cracks and pores began to appear on the surface of pectin, and the pores increased with increasing pressure (Figure 1B). Sugar beet pectin had a roughened pectin surface under a pressure of 350 MPa and was cracked or even destroyed when the pressure was 550 MPa, indicating that high pressure can degrade pectin (30). These results may be due to the decrease in Mw after different HPH treatments, resulting in the disruption of the nanostructure on the pectin surface (31).

### Effect of HPH on the Stability of NFC Orange Juice

#### Turbidity

**Figure 2:**
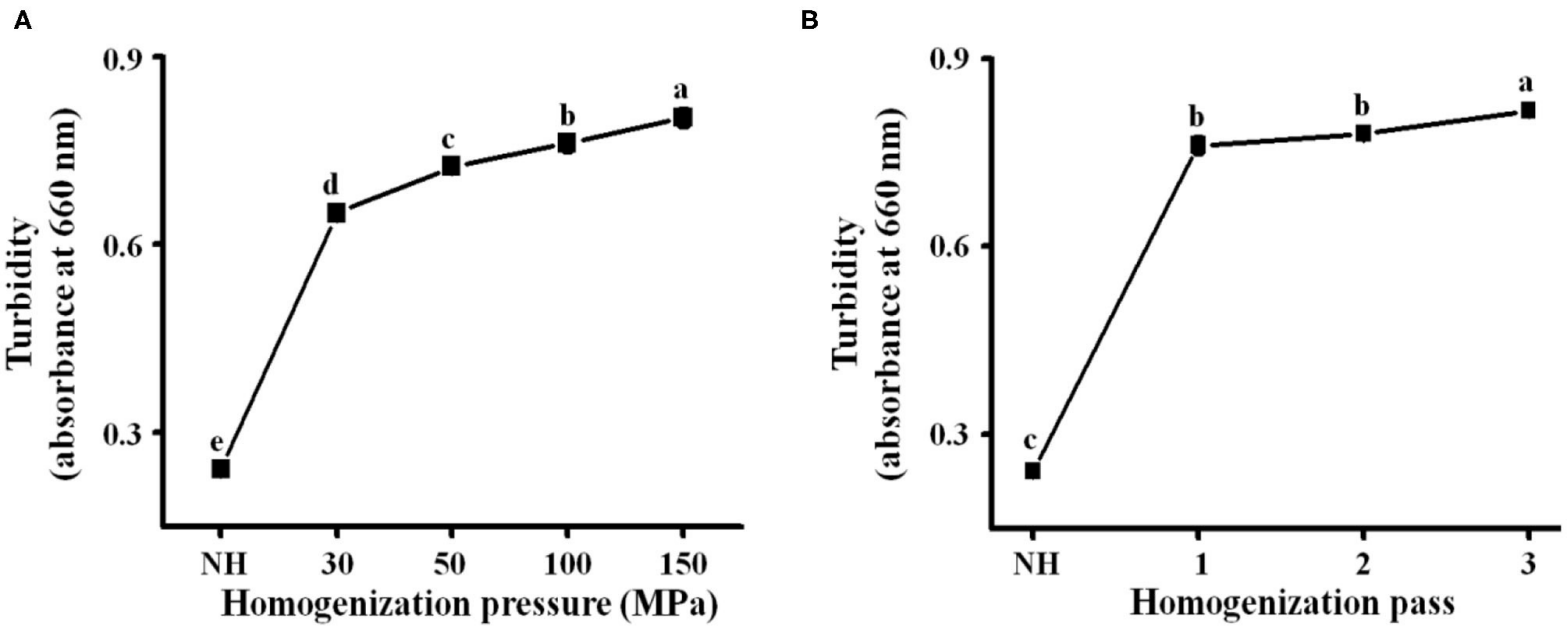
NFC orange juice was treated with different homogenization pressures (30, 50, 100, and 150 MPa) and numbers of homogenization passes (1, 2, and 3) to study the effect of HPH on the turbidity of NFC orange juice. **(A)** Homogenization pressure; **(B)** Homogenization pass.

Turbidity is an evaluation index of cloud stability (13), and high turbidity indicates good stability of the orange juice. As shown in Figure 2A, the turbidity of the homogenized orange juices [optical density at 660 nm (OD_660_), 0.65–0.82] was significantly (*P* < 0.05) higher than that of NH orange juice (OD_660_, 0.24). This is due to the enhancement of cavitation and the increase of shear rate caused by the increasing pressure, which caused stronger disruption of the NFC orange juice particles, making them more homogeneous (27). Turbidity increased from 0.78 to 0.82 when the number of passes was increased from 2 to 3 (Figure 2B). Subsequent homogenization passes caused further particle fragmentation (34). Interactions between the small particles and the serum phase may inhibit the production of precipitates (35, 36). Smaller particles tended to remain in suspension after centrifugation (which was always carried out at the same acceleration), increasing the absorbance values and turbidity (28). A previous report indicated that the increase of the OD_660_ value of orange juice after HPH was associated with improved stability of the orange juice (10, 37, 38). This confirms that the turbidity increases with increasing pressure and that HPH can effectively improve the stability of orange juice. Both homogenization pressure and homogenization pass treatments significantly increased the turbidity of orange juice (*P* < 0.05). Thus, HPH not only destroys particles but also binds them by altering the pectin structure in the serum phase, thereby increasing the turbidity and stability of NFC orange juice.

#### Pulp Sedimentation

**Figure 3:**
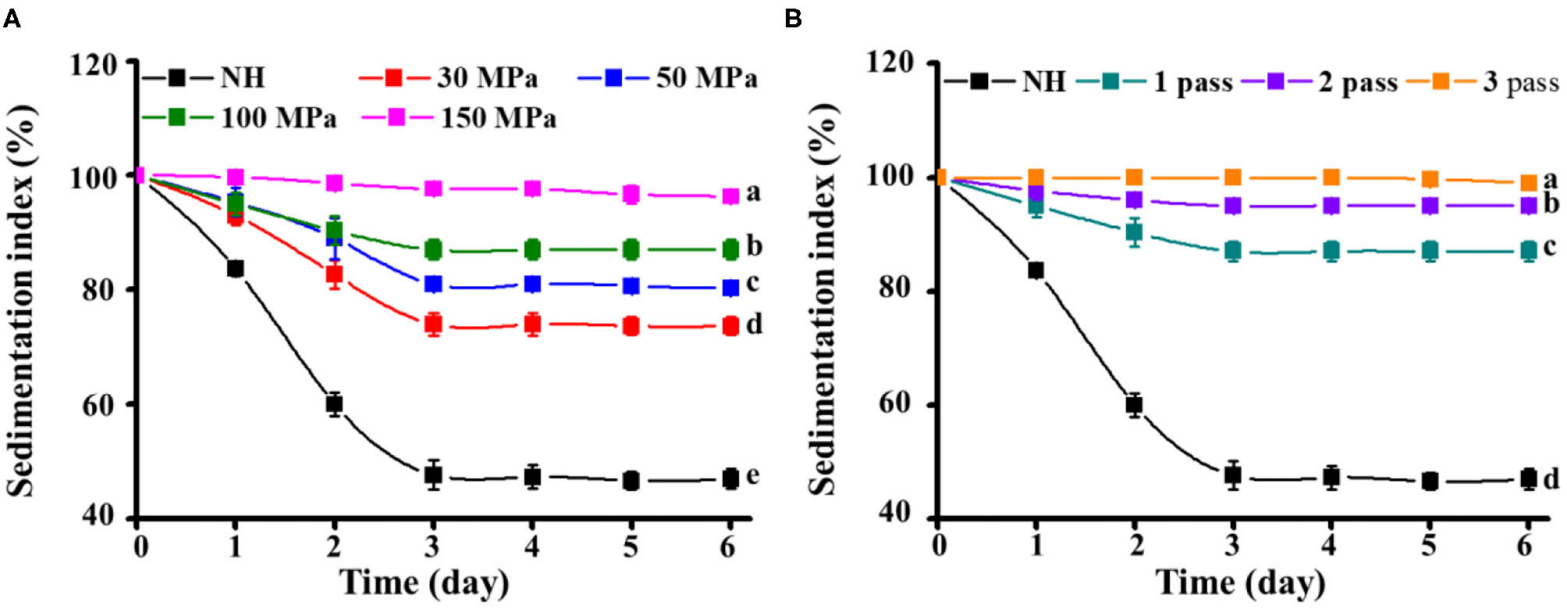
Effect of HPH treatments on the sedimentation index of NFC orange juice. **(A)** Homogenization pressure; **(B)** Homogenization pass.

Pulp sedimentation is a decisive factor in the cloud stability of NFC orange juice. A relatively high IS value represents a slow settling of particles in the orange juice, which provides better stability. Compared with homogenized orange juices (IS: 100% to 71–99%), NH orange juice (IS, 100% to 47%) showed faster sedimentation after 6 days (Figure 3), indicating that HPH can effectively inhibit the stratification of orange juice and improve its sensory characteristics. Compared with the homogenization pressure treatment, the number of homogenization passes made the particles in the orange juice more difficult to precipitate. The same phenomenon was observed in carrot juice, where higher pressure and more homogenization passes led to smaller changes in IS values, indicating that the juice became more stable. Stokes law describes the sedimentation velocity of spheres as a function of the properties of the particles and the dispersed medium (13). According to Stokes law, the particle sedimentation velocity is proportional to the particle size (diameter) and the difference between the densities of the particles and the dispersed medium (27). With relatively high pressure and more homogenization passes, NFC orange juice has more homogeneous particles. Therefore, the increased stability of homogenized orange juices may be related to the slower sedimentation due to the reduction in particle size during homogenization. In addition, due to the uniform size of the particles and large surface area, the resulting structure is quickly stabilized. Their settling was more difficult due to the drag of the opposing forces; therefore, the homogenized orange juices showed little change in IS values over 6 days. The stability of mixed juices (apple and kiwi) is influenced by particle size and interaction with the serum phase (39). HPH processing can release and alter serum phases, such as serum pectin, which binds to suspended solids and prevents precipitation, thereby increasing the stability of the cloud (40). Therefore, the combination of these particles and serum pectin also leads to minimal changes in IS values in homogenized orange juice. HPH is an important means of preventing pulp sedimentation.

#### Particle Size

**Figure 4:**
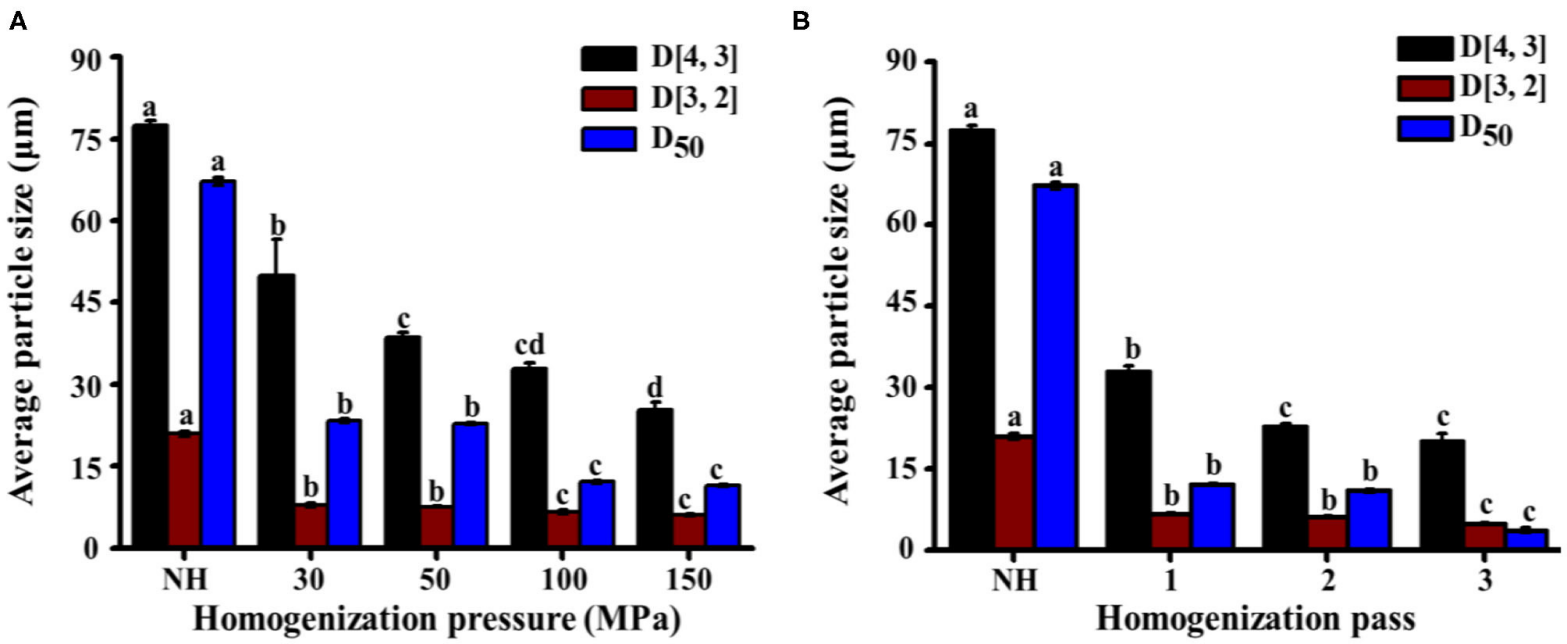
Effect of HPH treatments on the volume average particle size (D[4,3]), surface average particle size (D[3,2]), and average particle size (D_50_) of NFC orange juice. **(A)** Homogenization pressure; **(B)** Homogenization pass.

The particle diameters (D[4,3], D[3,2], and D_50_) of NH orange juice and homogenized orange juices are shown in Figure 4. D[4,3] is mainly influenced by large particles, while D[3,2] is more sensitive to small particles. These two indicators provide a complete picture of the system particle size (41). With increasing homogenization pressure and number of passes, D[4,3] and D[3,2] decreased simultaneously. These large and small particles had a similar effect on the HPH process. HPH reduced the particle diameter, which was consistent with previous observations for orange juice (10, 37) and other vegetable products such as tomato (36) and apple juices (42). At 30–50 MPa, D[4,3] decreased significantly (*P* < 0.05) from 49.9 μm to 39.6 μm, but the change of D[3,2] was not significant (*P* > 0.05) (Figure 4A). This indicates that in this pressure range, mainly the large particles were broken. When the pressure was further increased, D[4,3] did not change significantly (*P* > 0.05), but D[3,2] decreased significantly (*P* < 0.05) from 7.7 μm to 6.6 μm. High pressure was required to break up small-sized particles. The change in particle diameter between 100 MPa and 150 MPa was not distinct from that between 0 MPa and 50 MPa. The destructive effect of homogenizing pressure on suspended particles seemed to show an asymptotic behavior, with an increase in homogenizing pressure leading to smaller changes in particle size at higher pressures. This asymptotic behavior could be related to gap dimensions and shear stress since the stress required for disrupting smaller size particles becomes greater (20). When the number of passes increased from 2 to 3, the change in D[4,3] was not significant (*P* > 0.05), but D[3,2] decreased significantly (*P* < 0.05) from 6.1 to 5.0 μm (Figure 4B). This phenomenon indicated that there were no obvious changes in large-sized particles, but mainly an increase in small-sized particles. Furthermore, particle reduction can increase the brightness and improve the total color value of orange juice (19), in addition to increasing the accessibility of carotenoids and improving the sensory and nutritional properties of orange juice (43). The D_50_ of homogenized orange juices (3.7–12.1 μm) was significantly (*P* < 0.05) lower than that of NH orange juice (67.2 μm). The D_50_ tended to decrease with increasing pressure, but HPH treatment between 30 MPa and 50 MPa had no significant (*P* > 0.05) effect on D_50_. As the pressure continued to increase, the D_50_ decreased significantly (*P* < 0.05) from 22.8 to 12.1 μm, but continued pressure increases had no significant (*P* > 0.05) effect on D_50_. Further reduction of the cumulative particle size cannot be achieved by increasing the pressure beyond 100 MPa. As the number of passes increased, the D_50_ decreased significantly (*P* < 0.05) from 11.0 to 3.7 μm. HPH reduced the particle size in NFC orange juice, making it more homogeneous. This in turn increased the turbidity of the NFC orange juice and delayed pulp sedimentation, increasing the stability of the orange juice and slowing precipitation. It also improved the sensory and nutritional properties of orange juice and enhanced consumer acceptance.

#### Viscosity Curves

**Figure 5:**
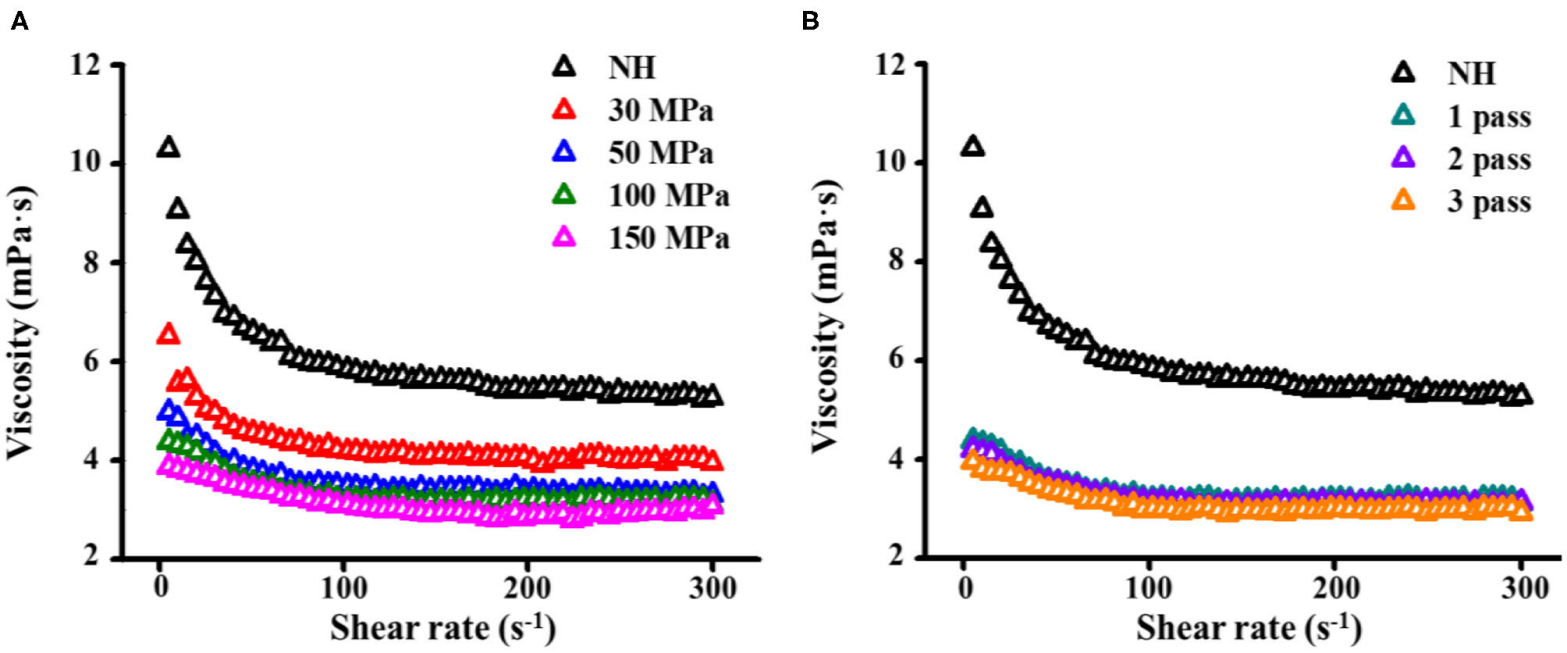
Effect of HPH treatments on viscosity curves of NFC orange juice. **(A)** Homogenization pressure; **(B)** Homogenization pass.

The apparent viscosity represents the resistance of the fluid to flow (18). With increasing shear rate, the shear stress of NFC orange juice increased and exhibited shear-thinning behavior, independent of treatment conditions (Figure 5). In contrast to NH orange juice, the viscosity of homogenized orange juices decreased gradually with increasing pressure and number of passes (Figure 5). The homogenization pressure treatment induced more viscosity decrease than the homogenization passes, and thereby reduced more resistance and energy consumption. These results were due to a reduction in particle size. The pectin solution treated with HPH showed reduced fluid viscosity (31). Increasing the pressure and number of passes depolymerizes the pectin side chains, thereby reducing their Mw and viscosity (44), which also reduces the viscosity of the orange juice. HPH alters the rheology of NFC orange juice by altering the size of macromolecules in the serum phase (45). Similarly, a decrease in juice viscosity after HPH has been reported in blended juice (21) and tomato puree (46). Since the viscosity of the fluid is related to frictional losses during processing, a reduction in the viscosity of NFC orange juice is desirable (18). HPH can reduce the viscosity of NFC orange juice by reducing the particle size and changing the size of the pectin molecules in the serum phase, thereby reducing fluid resistance and energy consumption in the processing industry.

#### PME Activity

**Figure 6:**
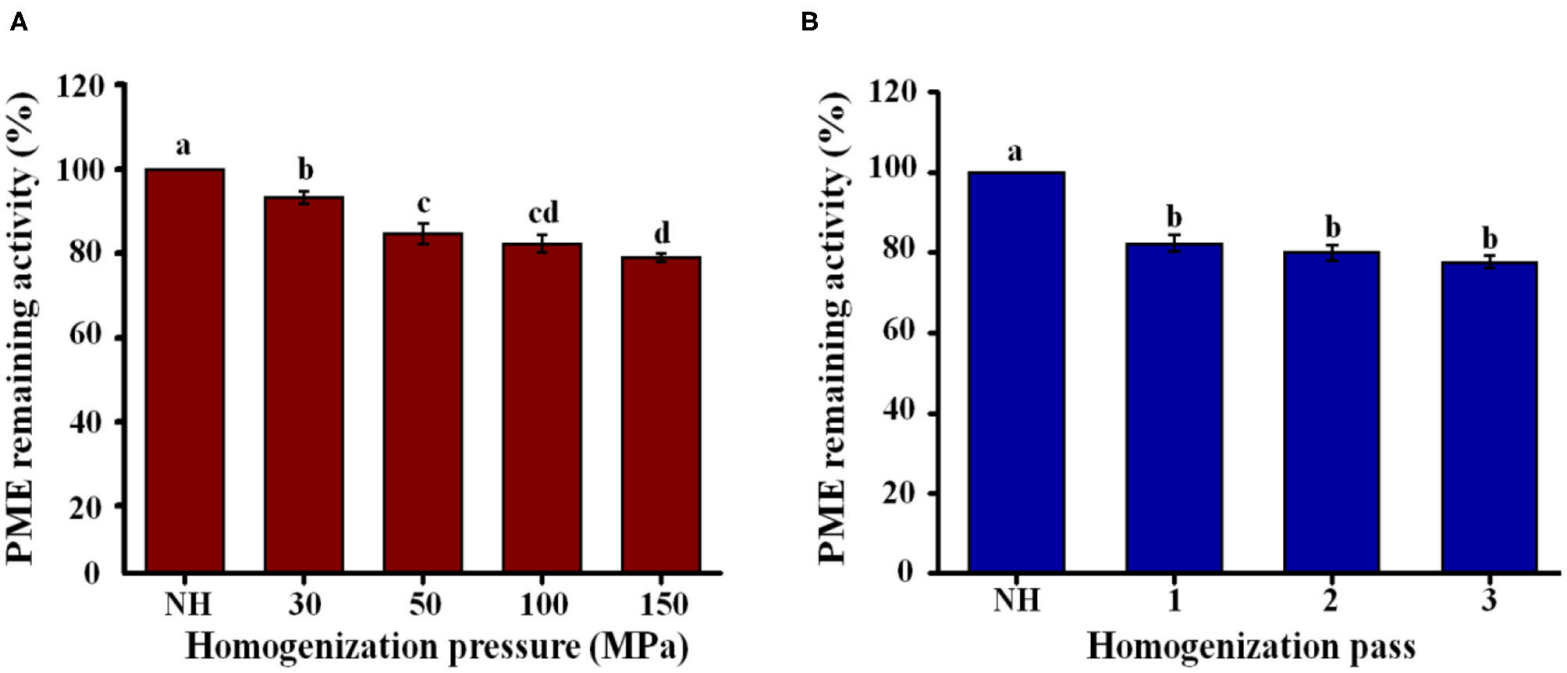
Effect of HPH treatments on PME activity of NFC orange juice. **(A)** Homogenization pressure; **(B)** Homogenization pass.

PME activity can promote orange juice clarification, negatively affect the quality of orange juice, and reduce consumer acceptability. Compared to NH orange juice, the PME activity of the homogenized orange juices decreased with increasing pressure, reaching a minimum value of 79% at 150 MPa (Figure 6A). When the number of passes increased from 1 to 3, there was no significant (*P* > 0.05) effect on the PME activity (77–82%) of the homogenized orange juices (Figure 6B). Compared with the homogenization pressure treatment, the activity of pectin methylesterase in orange juice was lower after more homogenization passes (Figure 6). The inactivation of PME activity prevents the loss of turbidity, increasing the commercial value of the orange juice. PME activity in orange juice decreased by only 20% after ultra-high-pressure homogenization (UHPH) for five passes at 170 MPa (47). After UHPH at 250 MPa with different inlet temperatures, the PME activity of the orange juice decreased by up to 38%, and the samples required five homogenization passes to achieve an 80% reduction (48). Although the PME activity of orange juice treated by HPH and UHPH was still residual, its stability was improved. This result may be due to the change of pectin structure, which made it easier to interact with particles in the serum phase, and thus inhibited precipitation. It is also possible that the particle size decreases due to HPH, thus improving stability. Therefore, it is necessary to further study the effect of HPH on pectin structure modification in NFC orange juice to explain how HPH improves the stability of NFC orange juice.

### Simulation Verification Experiment

**Figure 7:**
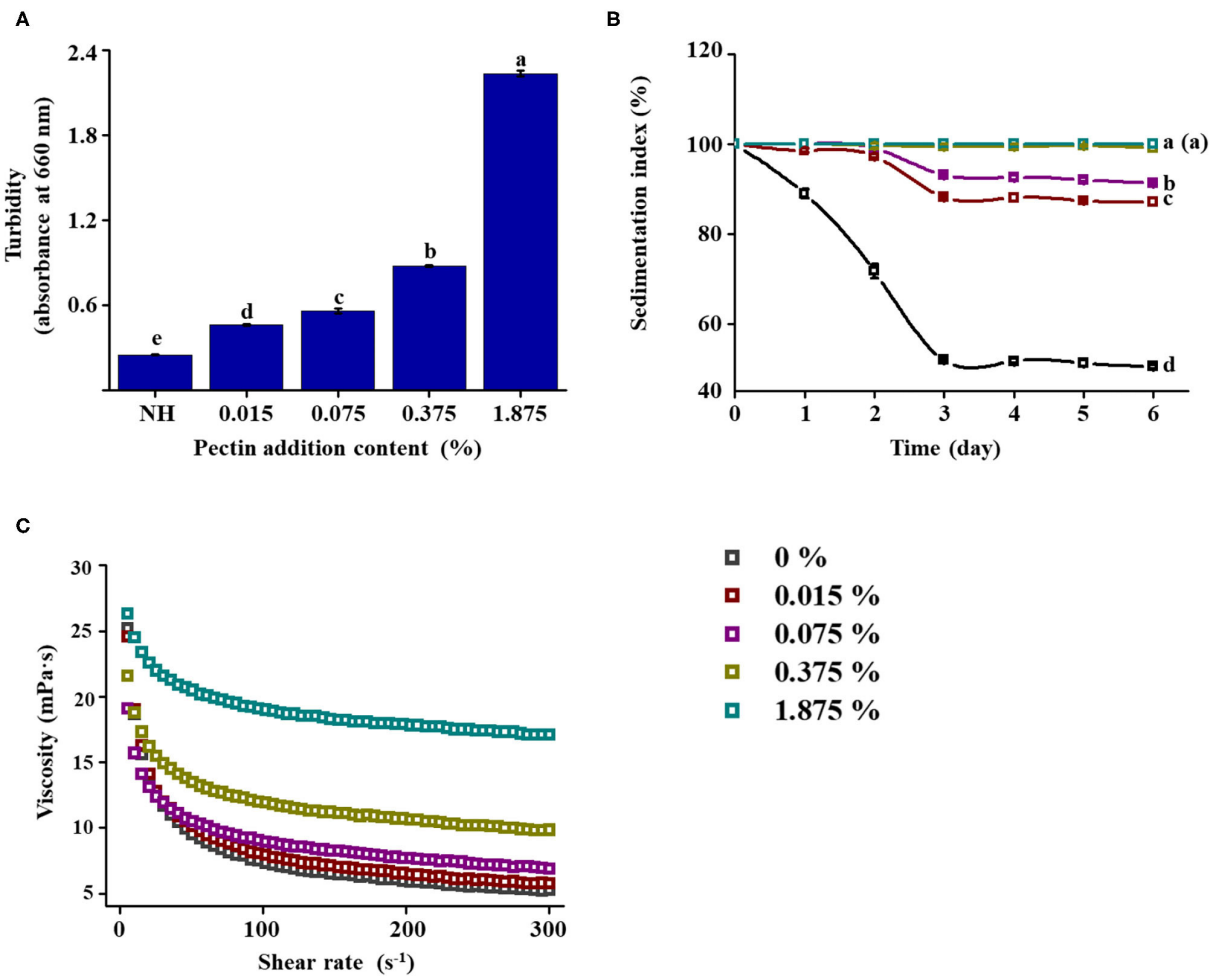
Effect of addition of pectin (after HPH) at various concentrations on the stability of NFC orange juice. **(A)** Turbidity; **(B)** Sedimentation index; **(C)** Viscosity curves.

Based on the above experiments, it was hypothesized that HPH could improve the stability of NFC orange juice by modifying the structural properties of pectin in NFC orange juice. The squeezed orange juice was directly filtered without HPH treatment, and the filtered orange juice was retained. At the same time, a pectin solution with a concentration of 0.075% was subjected to HPH treatment at 100 MPa, and different concentrations of pectin solution were added to the filtered NFC orange juice. The structure of the pectin used for backfilling was characterized, and its Mw and monosaccharide composition were determined to verify the consistency with previous results. The intuitive stability indicators such as turbidity, pulp sedimentation, and viscosity were measured to verify the effect of the structure-altered pectin on the stability of NFC orange juice. The Mw, linearity, and branching of pectin after 100 MPa treatment were similar those of pectin extracted from NFC orange juice under the same pressure treatment (Table 2). Compared with the non-pressure-treated pectin, the linearity increased and the RG contribution and branching decreased, which was consistent with our previous results. The stability of NFC orange juice was determined by adding different concentrations of homogenized pectin solution. With increasing pectin concentration, the OD_660_ value of NFC orange juice increased significantly (*P* < 0.05) from 0.25 to 2.24 (Figure 7A). As the pectin concentration increased, the sedimentation of particles was inhibited and the IS value of NFC orange juice increased from 39.6 to 99.9% after 6 days (Figure 7B). When the pectin concentration increased from 0 to 1.875%, the viscosity of NFC orange juice also increased (Figure 7C). The stability of NFC orange juice is improved by adding different concentrations of homogenized pectin. This further confirmed that the modification of the pectin structure affected the stability of NFC orange juice.

**Table 2:** Pectin structure properties (Mw and monosaccharide composition) after homogenization (100 MPa).

	**Before homogenization**	**After homogenization**	**In NFC orange juice before homogenization**
GalA (mol %)	60.57 ± 0.23^b^	71.63 ± 0.66^a^	72.86 ± 0.41^a^
Fuc (mol %)	0.10 ± 0.01^b^	0.18 ± 0.02^b^	0.24 ± 0.04^a^
Rha (mol %)	2.75 ± 0.36^a^	2.28 ± 0.24^b^	2.14 ± 0.06^b^
Ara (mol %)	13.48 ± 1.48^a^	6.87 ± 0.86^b^	8.26 ± 0.14^b^
Gal (mol %)	17.00 ± 0.49^a^	16.57 ± 0.34^a^	13.83 ± 0.31^b^
Glc (mol %)	5.31 ± 0.05^a^	1.83 ± 0.01^c^	2.27 ± 0.04^b^
Xyl (mol %)	0.79 ± 0.03^a^	0.64 ± 0.06^b^	0.42 ± 0.01^c^
GalA/(Gal+Ara+Rha)	1.82 ± 0.15^b^	2.79 ± 0.12^a^	3.01 ± 0.05^a^
Rha/GalA	0.0455 ± 0.0006^a^	0.0318 ± 0.0002^b^	0.0293 ± 0.0020^b^
(Gal+Ara)/Rha	11.08 ± 0.88^a^	10.29 ± 0.06^b^	10.34 ± 0.01^b^
Mw ( ×10^5^ Da)	9.17 ± 0.06^a^	2.54 ± 0.09^b^	2.62 ± 0.02^b^

*Different superscript letters indicate significant differences among groups (P < 0.05)*.

### Correlation Matrix Analysis

Correlations were used to describe the main variables of NFC orange juice stability and pectin structural properties after HPH treatment. Validation was performed by calculating the Pearson correlation coefficient [r] for each pairwise comparison (Table 3). The turbidity and IS of NFC orange juice were significantly positively (*P* < 0.01) correlated with the GalA content and linearity of pectin. The particle size and viscosity of NFC orange juice were significantly positively (*P* < 0.01) correlated with the Mw, RG contribution rate, and branching degree of pectin. Turbidity and IS were significantly (*P* < 0.01) negatively correlated with the RG contribution rate, branching degree, and Mw. There was a significant (*P* < 0.01) negative correlation between particle size and viscosity and pectin linearity. These relationships corroborate previous reports indicating that the improvement of NFC orange juice results from changes in pectin structure.

**Table 3:** Correlation matrix between stability and pectin characteristics in NFC orange juice.

	**Mw**	**GalA**	**GalA/(Gal+Ara+Rha)**	**Rha/GalA**	**(Gal+Ara)/Rha**
Turbidity	−0.967^**^	0.843^**^	0.958^**^	−0.959^**^	−0.941^**^
IS	−0.935^**^	0.953 ^**^	0.968^**^	−0.978^**^	−0.931^**^
D[4,3]	0.940^**^	−0.936^**^	−0.942^**^	0.958 ^**^	0.913^**^
D[3,2]	0.871^**^	−0.791^**^	−0.952^**^	0.961^**^	0.966^**^
D_50_	0.893^**^	−0.870^**^	−0.971^**^	0.962^**^	0.979^**^
Viscosity	0.953^**^	−0.859^**^	−0.912^**^	0.909^**^	0.887^**^

** *Indicates the significant correlations (P < 0.01)*.

## Conclusions

This work mainly investigated the effects of HPH on the structural modification of pectin, the cloud stability of NFC orange juice and their existing correlation relationship. The results showed that HPH could modify the structure of pectin in NFC orange juice and thus improve its stability. The improved stability could improve the sensory and nutritional properties of orange juice. With the increase of homogenization pressure and homogenization pass, HPH increased the GalA contents and linearity of pectin in NFC orange juice, while decreased the Mw, branching and RG contribution. At the same time, the smooth and compact surface of pectin appeared pores and cracks of different sizes, which also implied depolymerization of pectin. In addition, HPH reduced the particle sizes of orange juice and inhibited the sedimentation. Although the PME activity of NFC orange juice was still strong after HPH treatment, the cloud stability of NFC orange juice was improved. This was because the homogenized pectin interacted more easily with the particles in orange juice, thus effectively improving the stability of NFC orange juice. The stability index of NFC orange juice was strongly correlated with the structural properties of pectin, indicating that pectin directly or indirectly influenced the sensory qualities of orange juice. In this research, the relationship between pectin structure and stability of NFC orange juice was elucidated, which improved the sensory quality and market value of NFC orange juice, and improved the sensory and nutritional quality of NFC orange juice, increased consumer acceptance and commercial value, and provided theoretical guidance for the production of high-quality NFC orange juice.

## Data Availability Statement

All datasets generated for this study are included in the article material.

## Author Contributions

WY: methodology, investigation, conducting experiments, and writing—original draft. JC: methodology and formal analysis. SZ: validation and formal analysis. LF: formal analysis. YW: investigation. JL: supervision and writing—review and editing, and funding access and support. JZ: formal analysis, conceptualization, supervision, writing—review and editing, and funding access and support. All authors contributed to manuscript revision and read and approved the submitted version.

## Conflict of Interest

The authors declare that this study received funding from CHENGUANG Biotech Group Co., Ltd., NESTLE R&D Ltd. (China), and FRUITOPS Co., Ltd. The funders were not involved in the study design, collection, analysis, interpretation of data, the writing of this article and the decision to submit it for publication.
